# A hybrid attention multi-scale fusion network for real-time semantic segmentation

**DOI:** 10.1038/s41598-024-84685-6

**Published:** 2025-01-06

**Authors:** Baofeng Ye, Renzheng Xue, Qianlong Wu

**Affiliations:** 1 School of Computer and Control Engineering, Qiqihar University, Qiqihar, 161003 China; 2 Heilongjiang Key Laboratory of Big Data Network Security Detection and Analysis, Qiqihar University, Qiqihar, 161000 China

**Keywords:** Semantic segmentation, Real-time processing, Attention mechanism, Edge detection, Receptive field, Computational science, Computer science

## Abstract

In semantic segmentation research, spatial information and receptive fields are essential. However, currently, most algorithms focus on acquiring semantic information and lose a significant amount of spatial information, leading to a significant decrease in accuracy despite improving real-time inference speed. This paper proposes a new method to address this issue. Specifically, we have designed a new module (HFRM) that combines channel attention and spatial attention to retrieve the spatial information lost during downsampling and enhance object classification accuracy. Regarding fusing spatial and semantic information, we have designed a new module (HFFM) to merge features of two different levels more effectively and capture a larger receptive field through an attention mechanism. Additionally, edge detection methods have been incorporated to enhance the extraction of boundary information. Experimental results demonstrate that for an input size of 512 × 1024, our proposed method achieves 73.6% mIoU at 176 frames per second (FPS) on the Cityscapes dataset and 70.0% mIoU at 146 FPS on Camvid. Compared to existing networks, our Model achieves faster inference speed while maintaining accuracy, enhancing its practicality.

## Introduction

Semantic segmentation is a typical task in computer vision, where the goal is to classify each pixel in an image, allowing pixels with the same label to share common visual properties. It has wide applications in agriculture, industrial automation, autonomous driving, and medical diagnosis. Regarding drones, identifying areas where drones can fly and flight paths to ensure drones do not fly into other regions requires both accuracy and efficient inference speed.

A large number of researchers have made significant progress in the field of semantic segmentation to meet the needs of various applications. The first proposed semantic segmentation network was FCN^1^, based on the convolutional neural network VGG^2^. It obtains features through convolution, pooling, and other operations on images. However, because it ignores spatial information, the obtained feature maps are relatively blurry, especially at the boundary. Therefore, the segmentation accuracy of fully convolutional neural networks is low. ENet^3^ improves real-time inference speed by reducing the size of images and modifying the network structure, making the Model more concise. However, this also leads to a lack of perceptual domain and poor recognition ability for large objects. In the initial stages, most models sacrifice spatial details to improve inference speed, resulting in unsatisfactory application performance.

In some complex scenes, the existing single visual mode to detect small targets has limitations, especially in some complex conditions, the accuracy will be greatly affected^4–6^. A special research method is proposed for small target detection^6^. By associating motion patterns with semantics^4^. The proposed SSTNet achieves the highest accuracy in infrared dim target missions^5^. The proposed LASNet can extract features of small targets more comprehensively. Each of the above three approaches presents strong advantages.Their proposed method provides a reference for the detection and segmentation of some small objects on the Cityscape dataset.

**Fig. 1:**
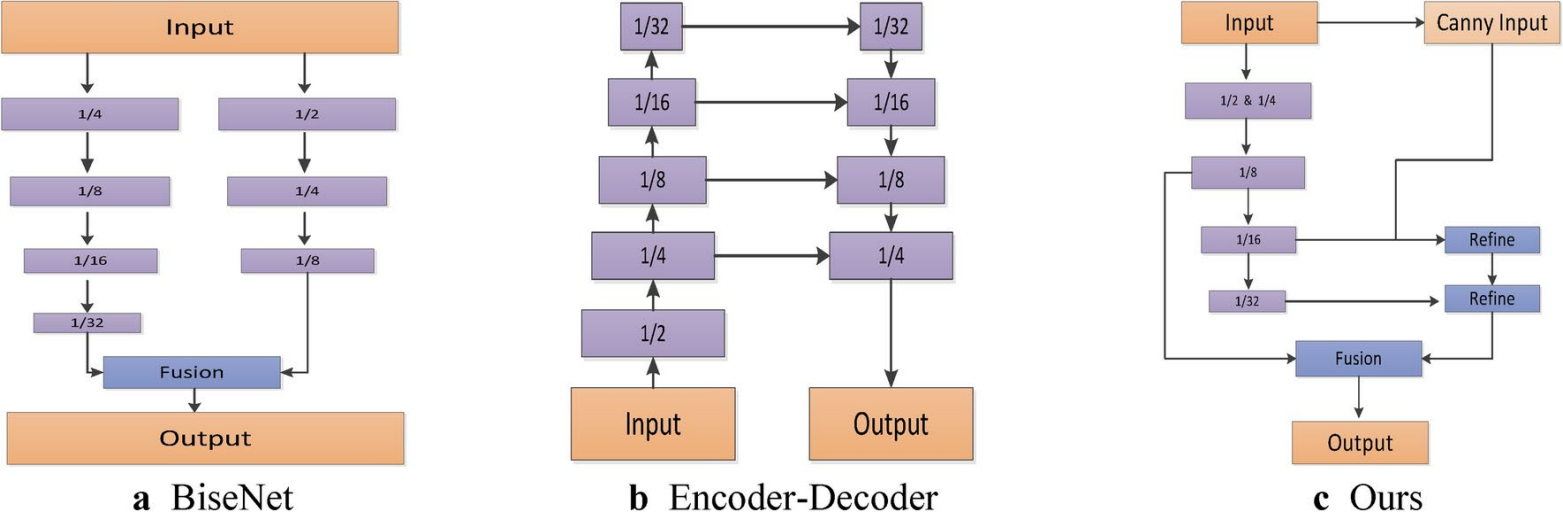
Illustration of the architectures BiSeNet, encoder-decoder and our proposed approach. (**a**) denotes the BiSeNet structure. (**b**) indicates the Encoder-Decoder architecture. (**c**) indicates our proposed architecture, which eliminates the time-consuming additional paths. It uses Canny edge detection and feature refinement to strengthen the trained feature maps. Among them, violet boxes in a, b and c all use Conv module to downsample pictures.

Many researchers have started using U-shaped structures to minimize spatial loss, As shown in (Fig. 1b). Encoders typically serve as the backbone for pre-training in semantic segmentation tasks, extracting high-level semantic information through repeated upsampling. However, the encoder-decoder architecture is primarily designed for traditional semantic segmentation tasks, and the granularity of the decoder remains insufficient, leading to the loss of edge information in segmentation results. Addressing these issues, BiSeNet^7^, As shown in (Fig. 1a) proposes the feature fusion module (FFM), which integrates features from different levels by connecting features from two paths and then normalizing them to achieve more effective fusion. Compared with other models, the U-shaped architecture still has certain deficiencies in segmentation accuracy and other aspects. Therefore, simultaneously achieving high efficiency and high precision and designing an architecture tailored explicitly for real-time semantic segmentation tasks is crucial.

Yu et al., who have made milestone progress, proposed a bilateral segmentation network with spatial path (SP) and context path (CP) for real-time semantic segmentation tasks. After that, excellent works such as RTFormer^8^ and DDRNet^9^ appeared, which also processed low-resolution feature maps separately from high-resolution feature maps, and finally improved real-time performance through effective fusion. Low-level spatial and high-level semantic information are crucial in semantic segmentation tasks. The design of SP reduces spatial information loss, while CP extracts more comprehensive semantic information. Due to the design of these two paths, we can separately handle spatial and semantic information. However, the authors of STDC Net posit that utilizing a backbone in BiSeNet, predicated on existing image classification models, was not explicitly tailored for semantic segmentation tasks and may consequently impact segmentation accuracy. As a result, they devised the STDC module to rectify this issue within BiSeNet’s architecture, thereby yielding enhanced segmentation performance.

Therefore, we propose an improved real-time semantic segmentation network. Firstly, we remove the spatial path (SP), attention refinement module (ARM), and feature fusion module (FFM) from the original architecture of BiSeNet and adopt the backbone designed in STDC Net^10^. The initial stages are designed to be wide-channel shallow layers to capture spatial information and narrow-channel deep layers to capture semantic information. Then, we design two new modules and incorporate edge detection methods. Figure 1c illustrates the architecture of our designed Model. Inspired by spatial attention^11^ and channel attention^12^, we devise hybrid feature refinement module (HFRM) and hybrid feature fusion module (HFFM). The attention mechanism captures long-range semantic information, enhancing understanding of feature maps. We obtain better feature information through the design of the HFRM module. Additionally, considering that most semantic segmentation methods overlook edge information, we integrate edge detection methods to enhance feature representation for improved segmentation accuracy. Extensive experimental evaluations demonstrate the effectiveness of the two new modules we designed.

Our main contributions are summarized as follows:

1. We design two new modules that can effectively fuse and improve feature maps for channel attention and spatial attention concepts. These two modules require less computing resources and significantly improve the performance of our model.
2. We assist the training by adding a traditional edge detection algorithm. Since more edge information is added, the feature representation is effectively enhanced.
3. Our proposed method has achieved outstanding results in many experiments. Experimental results indicate that the algorithm proposed in this paper achieves excellent performance. Running at a speed of 176.3 FPS on an NVIDIA GTX 1080Ti card, it achieves a mIoU of 73.6% on the cityscape dataset.

## Related work

In recent years, with the rapid development of artificial intelligence technology, there have been many excellent models in computer vision, and there has been significant improvement in semantic segmentation. These outstanding frameworks have provided many references, from FCN to the current BiSeNet^7^. This section introduces three parts: general semantic segmentation, real-time semantic segmentation, and lightweight frameworks.

### General semantic segmentation

Traditional semantic segmentation algorithms differ significantly from current algorithmic approaches. They mainly use low-level features such as grayscale, RGB, and textures to segment images, employing threshold-based selection, pixel clustering-based segmentation, and graph partitioning segmentation methods. With the emergence and development of deep learning, the introduction of early networks such as AlexNet^13^, VGG^2^, and ResNet^14^ laid the foundation for semantic segmentation. The introduction of UNet^15^ formally propelled semantic segmentation into a stage of rapid development, and UNet has achieved outstanding results in various tests. SegNet^16^ consists of an encoder network, a corresponding decoder network, and a pixel-wise classification layer to restore high-resolution feature maps. RefineNet^17^ can be divided into two pathways, downward and upward, and employs numerous residual connections to generate high-resolution semantic feature maps. Mask2Former^18^ is a general-purpose structure for image segmentation that differs from other specialized structures and uses masking attention in the Transformer decoder to improve performance. However, a larger scale often accompanies the complexity of most model frameworks, and more model parameters mean higher memory requirements and lead to increased computing costs. Limit their usefulness in resource-limited scenarios.

### Real-time semantic segmentation

Currently, real-time semantic segmentation models are being applied in many practical applications, and the attention to real-time semantic segmentation is growing. In 2016, researchers first proposed the ENet^3^ model for real-time semantic segmentation, a milestone in this field. ContextNet^19^ employs pruning techniques to accelerate model segmentation. ERFNet^20^ maintains high efficiency and accuracy using residual connections and decomposed convolution layers. LEDNet^21^, while adopting the ENet structure, utilizes ResNet as the backbone encoder and employs channel shuffling on each residual block to improve model inference speed. NDNet achieves faster inference speeds by pruning redundant information and using pointwise convolutions through feature map connections. FPENet^22^ encodes context at multiple scales in the encoder using multiple FPE blocks, each composed of convolutions extended to different depths to strike a balance between accuracy and speed.

In recent years, with excellent CV performance, many excellent methods have emerged to promote real-time semantic segmentation development greatly. One representative is ViT^23^. Experiments have proved that it is successful to introduce a transformer into CV, but it is challenging to meet the real-time requirements due to the high computing cost of attention. To solve this problem, researchers have designed several excellent models. SeaFormer^24^, for example, whose main innovation is the design of a plug-and-play universal attention module. Finally, it performs well on semantic segmentation and its downstream tasks. RTformer^8^ proposed a set of high-real-time semantic segmentation schemes based on Transform. The authors adopt a GPU-friendly attention module in the low-resolution branch to capture high-level global context information. In contrast, the authors introduce a cross-resolution attention mechanism for processing in the high-resolution branch, achieving a balance between precision and speed.

While these methods balance accuracy and speed, they sacrifice lower-level details and overlook the importance of edge regions. Moreover, the research on real-time performance needs to be improved. In our Model, we simultaneously consider low-level details and high-level semantics to achieve high efficiency and precision.

### Lightweight framework

U-Net, as a classical model of image segmentation, has been studied by researchers. At present, improvements to U-Net are also trying to achieve higher accuracy with less computation. For example^25^, compatible with Conv by integrating KAN.With the emergence and application of deep convolutional and separable convolutions, lightweight architecture models have begun to develop rapidly. Among them are Xception^26^, LadderNet^27^, ShuffleSeg^28^, etc., which have achieved a decent balance between speed and accuracy in image processing tasks. The core idea under the lightweight backbone network is to enhance speed while maintaining accuracy as much as possible. MobileNet V2^29^ employs pointwise convolutions and deep convolutions for feature extraction, which can support good network performance while being more lightweight. These studies aim to reduce the Model’s parameter count without compromising accuracy. However, too few parameters can lead to a decrease in model performance. Considering computational complexity, real-time inference scenarios, and adjusting the backbone networks for different tasks, we have designed an efficient real-time semantic segmentation model.

## Our approach

Our framework selects STDC 2 from STDC Net^10^ as the encoder in this paper. Based on BiSeNet^7^, we redesigned two attention mechanism modules to fuse feature maps from different levels effectively. Additionally, we introduced edge detection methods into the Model to improve edge detection in images. In this section, we first introduce the hybrid feature refinement module and the hybrid feature fusion module. Then, we provide an overview of the training process, concluding with a discussion of the overall framework.

### Proposed hybrid feature refinement module

We observe that semantic information in images forms the basis of semantic segmentation, requiring a larger receptive field to acquire more comprehensive semantic information. Attention modules can capture semantic information over longer distances, enhancing the understanding of feature maps. BiSeNet^7^ utilizes attention mechanisms, designing the attention refinement module (ARM) to capture background information^30^. While proposing a new model, attention mechanisms are also added to identify low-contrast capillary regions.

For example, the GCN-DE proposed by^31^ not only improves the segmentation accuracy, but also reduces the complexity by forwarding deep features into an efficient global association model, and then calculates the near and far dependencies respectively to meet the real-time requirements^32^. The proposed GSC module completes the semantic mismatch by generating illusion nodes, which can supplement semantic information more quickly and comprehensively.

However, it only focuses on channel information^12^ while neglecting spatial information^11^. We cannot solely focus on “what” but also need to emphasize “where”.

For example, both the methods proposed by DANet and CBAM combine channel attention and spatial attention, and both have achieved excellent results. However, DANet is difficult to apply in practice due to its high computational amount, so designing a lightweight attention mechanism is very effective. However, CBAM attention mechanism is fixed, that is, channel attention and spatial attention are independently and sequentially executed. This design may not be able to accommodate all types of tasks and data sets, and may sometimes require more flexible attention mechanisms. Compared with the module complexity proposed by DANet and Non-Local Net, the HFRM module designed by us belongs to the linear level and has a complexity of *O(n)*. It can better meet the requirements of real-time. In the case of small data sets or uneven data distribution, CBAM may lead to overfitting of the model. This is because attention mechanisms may focus too much on certain features and neglect other important information. We use edge detection to assist HFRM to obtain more features.

**Fig. 2:**
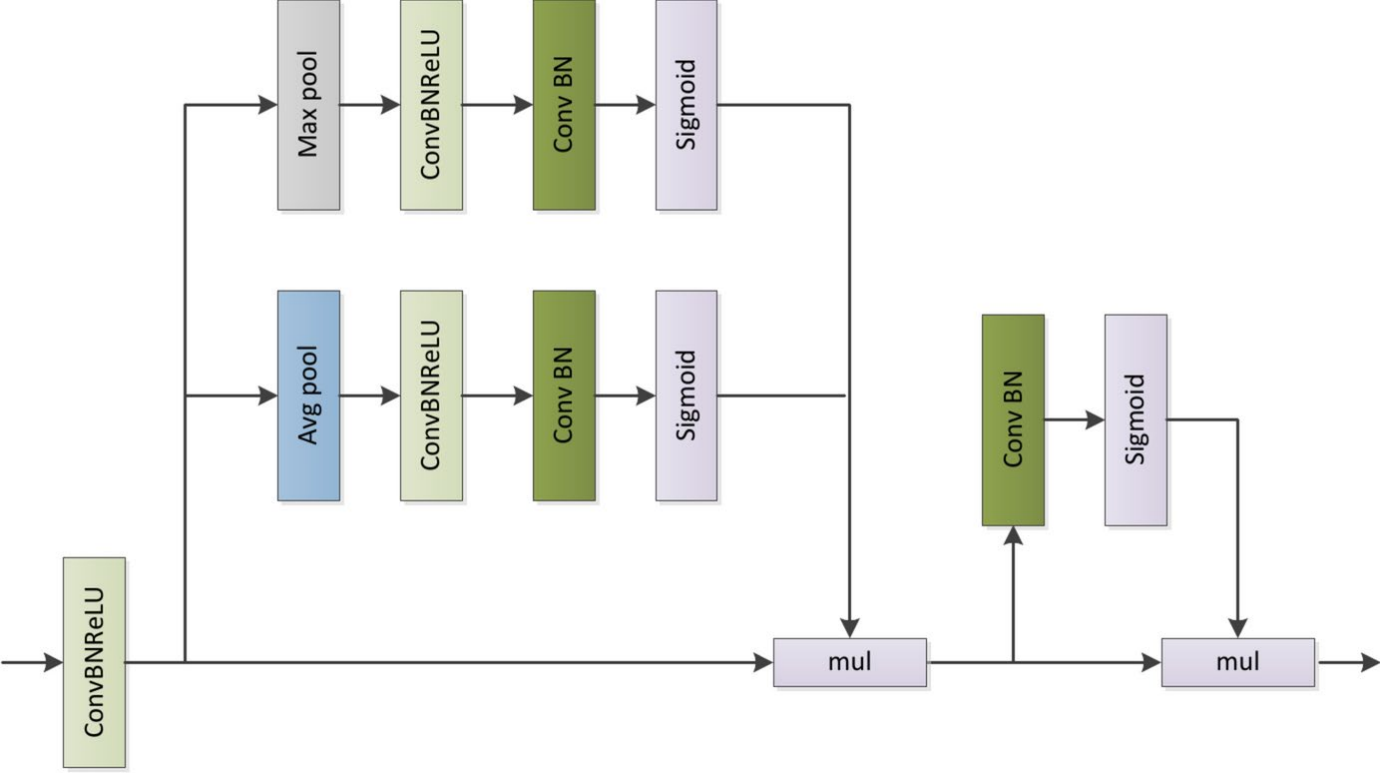
The detailed architecture of modules we propose components of the hybrid feature refinement module (HFRM).Where mul is multiplicative element by element.

Therefore, we should also consider lightweight when combining channel attention and spatial attention. As shown in Fig. 2, The hybrid feature refine module (HFRM) processes the input feature map with ConvBNReLU. This way can ensure that the application of the ReLU function after the BN layer can ensure that the output value does not appear too many zero values, and the data has been normalized before entering the ReLU. The ReLU function can help the model converge faster due to its properties. This process can be further accelerated when combined with BN layers, which help to improve the direction of gradient descent and make the learning process more efficient. After that, we will perform maximum pooling and average pooling on the feature maps to generate two C × 1 × 1 feature maps. The pooling operation can reduce the size of the feature map, thereby reducing the computational complexity. The maximum pooling operation selects the maximum value in the local area as the output, which retains the most prominent and important features in the image. The average pooling operation obtains the overall features of the local area by selecting the average value. Through this collocation, we obtain more obvious feature information. Although some information will be lost to a certain extent, we believe that this processing is worth it. After pooling operation, we perform ConvBNReLU operation and ConvBN operation. ks of ConvBNReLU is selected as 3, ks of ConvBN is selected as 1, and 1 × 1 conv is used to avoid the problem that the input must be fixed in scale and reduce the amount of calculation. A Sigmoid activation function is applied to generate channel attention weights. These weights will be applied to each channel of the original feature map, then, this feature map is processed through a ConvBN to generate spatial attention weights, and finally, the attention vector is calculated using Sigmoid to guide the model feature learning. We finally combine channel attention with spatial attention through the “mul” operation, which helps to enhance the feature representation of different channels, while spatial attention helps to extract key information at different locations in space. While refining the feature maps, HFRM can combine the context information captured in the backbone network with the global context information. Since there is no upsampling operation in the module, the pooling operation also effectively reduces the amount of computation and memory consumption, so the computational cost of HFRM is very low.

### Proposed hybrid feature fusion module

**Fig. 3:**
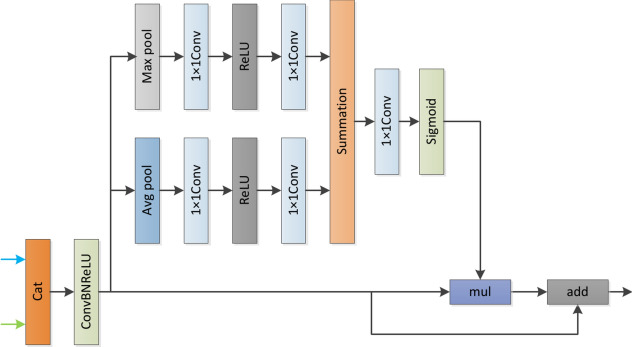
The detailed architecture of modules we propose components of the hybrid feature fusion module (HFFM).Where mul is multiplicative element by element.

In semantic segmentation, feature fusion can capture multi-scale information, combine shallow local information and edge information with deep global and semantic information, so that the model can better process complex image content. A single feature extractor or network layer may not be able to fully capture all the important information. Feature fusion can make up for this deficiency and reduce information loss. For example, ANNNet, DDRNet and BiseNet V3 have feature fusion modules. The fusion module designed by DDRNet is not plug and play and has great limitations. ANNNet only pays attention to spatial attention, but neglects channel attention. As shown in Fig. 3, arrows of different colours represent feature maps from two different levels. These characteristics of the decoder capture rich semantic information and retain rich spatial information. In other words, spatial information is low-level, while semantic information is high-level, and feature maps from different levels cannot be directly concatenated. Therefore, a feature fusion module must be designed to merge these features. Therefore, we propose a plug and play attention mechanism with space and channel integration, and does not require a high amount of computation.

We run the “Cat” operation on two different feature maps to solve the feature mismatch problem and get more feature information. ConvBNReLU processing is then performed to avoid non-normalized features^33^.Then Then average pooling and maximum pooling operations are carried out respectively to obtain feature maps with different scale context information. Then, while keeping the size of the feature map unchanged, the feature map is processed by k = 1 convolution layer, which can increase the nonlinearity of the model and improve the generalization ability of the model. We reduce the output channel by 4 times, effectively reduce the number of parameters and computational complexity, and improve the computational efficiency. We use Relu as the activation function because Relu is less computative and some neurons have an output of 0, which makes the network sparse and reduces parameter interdependence. Finally, the use of 1 × 1 conv avoids the problem that the input must be fixed size and reduces the amount of computation. Finally, the attention vector is generated using Sigmoid. We multiply the attention vector by the original feature map.The attention mechanism and residual feature are applied to feature fusion, and a dynamic feature fusion method is designed.

### Proposed edge detection

Edge detection is used to capture and learn edge information in images. Compared to semantic segmentation tasks, edge detection is Lightweight, only requiring extraction and identification of object edges, thus commonly used to improve segmentation performance. To assist the Hybrid Feature Refinement Module, we use the Canny operator^34^, which belongs to traditional edge detection algorithms, mainly targeting parts of the object edges that are blurry or inaccurately segmented. The features obtained through edge detection are then connected with the semantic features extracted from the backbone. Since the Canny operator utilizes a multi-step algorithmic process and more complex parameter settings, mainly focusing on Gaussian filtering and gradient calculation steps, using the Canny operator at each stage increases computational overhead significantly. After multiple experiments, we ultimately used feature maps with input sizes of 1/8 and 1/16. The final choice for the threshold of the Canny operator was^31^.

We consider edge detection assistance a weighted method similar to an attention mechanism. The feature map will be a binary classification representation for the final output edges of the feature map, controlled by gradient and threshold. We capture edge feature maps and multiply the classification results of edge detection by parameters determined by the results of the segmentation head. Additionally, different scale feature representations can be captured by detecting edges at various scales. The support for edge detection is as follows:

1 $${\varvec{F}}={\sum }_{{\varvec{j}}=1}^{{\varvec{H}}}{\sum }_{{\varvec{i}}=1}^{{\varvec{W}}}{{\varvec{F}}}_{{\varvec{i}}{\varvec{j}}}\times \boldsymbol{\alpha }$$

where F is the edge detection feature map, and α is the edge detection parameter.

Due to significantly fewer edge pixels than non-edge pixels in street scenes, this falls under the category of class imbalance problem. We jointly optimize edge learning using binary cross-entropy loss function and dice loss. Dice Loss is primarily used to measure the overlap between predicted results and ground truth. Since it is insensitive to the number of foreground/background pixels, it can moderately alleviate the class imbalance problem. The loss calculation for edge prediction results is as follows:

2 $${{\varvec{L}}}_{{\varvec{t}}{\varvec{o}}{\varvec{t}}{\varvec{a}}{\varvec{l}}}({\varvec{O}},{\varvec{T}})={{\varvec{L}}}_{{\varvec{d}}{\varvec{i}}{\varvec{c}}{\varvec{e}}}({\varvec{O}},{\varvec{T}})+{{\varvec{L}}}_{{\varvec{b}}{\varvec{c}}{\varvec{e}}}({\varvec{O}},{\varvec{T}})$$

where *O* $\in$ ${R}^{H\times W}$ represents the predicted edge output image, *T* $\in$ ${R}^{H\times W}$ Represents the corresponding edge target image. ${L}_{dice}$ denotes the dice loss and ${L}_{bce}$ Denotes the binary cross-entropy loss. The loss functions are given as follows:

3 $${{\varvec{L}}}_{{\varvec{d}}{\varvec{i}}{\varvec{c}}{\varvec{e}}}({\varvec{O}},{\varvec{T}})=1-\frac{2\sum_{{\varvec{i}}}^{{\varvec{H}}\times {\varvec{W}}}{{\varvec{O}}}^{{\varvec{i}}}{{\varvec{T}}}^{{\varvec{i}}}+{\varvec{\varepsilon}}}{{\sum }_{{\varvec{i}}}^{{\varvec{H}}\times {\varvec{W}}}{({{\varvec{O}}}^{{\varvec{i}}})}^{2} +{\sum }_{{\varvec{i}}}^{{\varvec{H}}\times {\varvec{W}}}{({{\varvec{T}}}^{{\varvec{i}}})}^{2}+{\varvec{\varepsilon}}}$$

4 $${{\varvec{L}}}_{{\varvec{b}}{\varvec{c}}{\varvec{e}}}({\varvec{O}},{\varvec{T}})=-\frac{1}{{\varvec{H}}\times {\varvec{W}}}(({{\varvec{T}}}^{{\varvec{i}}}*{\varvec{l}}{\varvec{o}}{\varvec{g}}({{\varvec{O}}}^{{\varvec{i}}}))+(1-{{\varvec{T}}}^{{\varvec{i}}})*{\varvec{l}}{\varvec{o}}{\varvec{g}}(1-{{\varvec{O}}}^{{\varvec{i}}})$$

where i represents the i-th pixel, set to 1, this is a parameter to avoid division by zero. The subsequent experimental section demonstrates that the edge detection method can effectively enhance feature representation.

### Network architecture

**Fig. 4:**
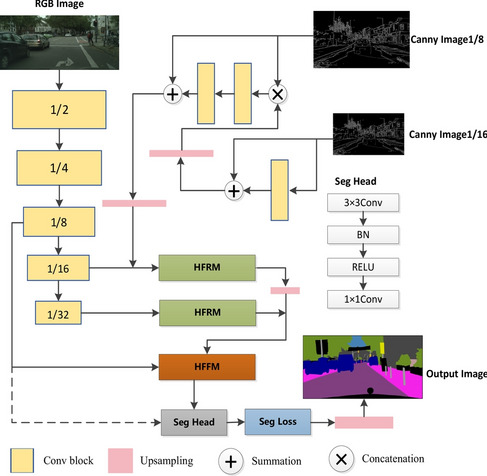
Framework description: Seg-head represents the segmentation head, consisting of a 3 $\times$ 3 Conv-batchnorm-ReLU layer followed by a 1 $\times$ 1 Conv layer. The output dimension of the segmentation head is the number of classes.

Based on the techniques and edge detection methods using HFRM, we propose a lightweight segmentation network for real-time segmentation. The specific details are shown in (Fig. 4). We have reexamined and redesigned feature refinement and fusion, integrating new technologies. We have selected STDC 2 as the backbone of the Model. The Conv block in the figure uses Conv-BN-ReLU to extract 1/2, 1/4, 1/8, 1/16, and 1/32 features. Convolution operations with kernel size 33 and stride of 2 are used at 1/2 and 1/4. After a convolution operation with kernel size 33 and stride 2 at 1/8, 1/16, and 1/32, a convolution operation with kernel size 33 and stride 1 is performed. For convolution operations of Canny images, we use convolution operations with kernel size of 33 and stride of 1. The Canny operator is employed for edge detection input, encoded and linked to the feature maps captured by the backbone. HFRM is utilized for smaller resolution feature maps to capture superior features, while HFFM merges the downsampled 1/8 feature maps with the advanced features generated by HFRM. We share the encoder at the front end, significantly boosting speed. In our design, edge detection aids in refining high-level feature maps with HFRM. HFFM combines low-level feature maps with spatial information and high-level feature maps with semantic information.

### Seg head module

Our Model’s design for seg head is 3 × 3 Conv-BN-ReLU followed by a 1 $\times$ 1 conv. Utilizing Conv-BN-ReLU, features can be effectively extracted and spatial information can be retained. 1 $\times$ 1 Conv operation reduces the computational load and memory consumption of subsequent operations by reducing the number of channels of feature graphs, especially when large-size images or large amounts of data need to be processed. We can adjust the segmentation head computational complexity by controlling the channel dimension C to improve the feature representation without additional inference costs to ensure real-time.

## Experimental result

**Fig. 5:**
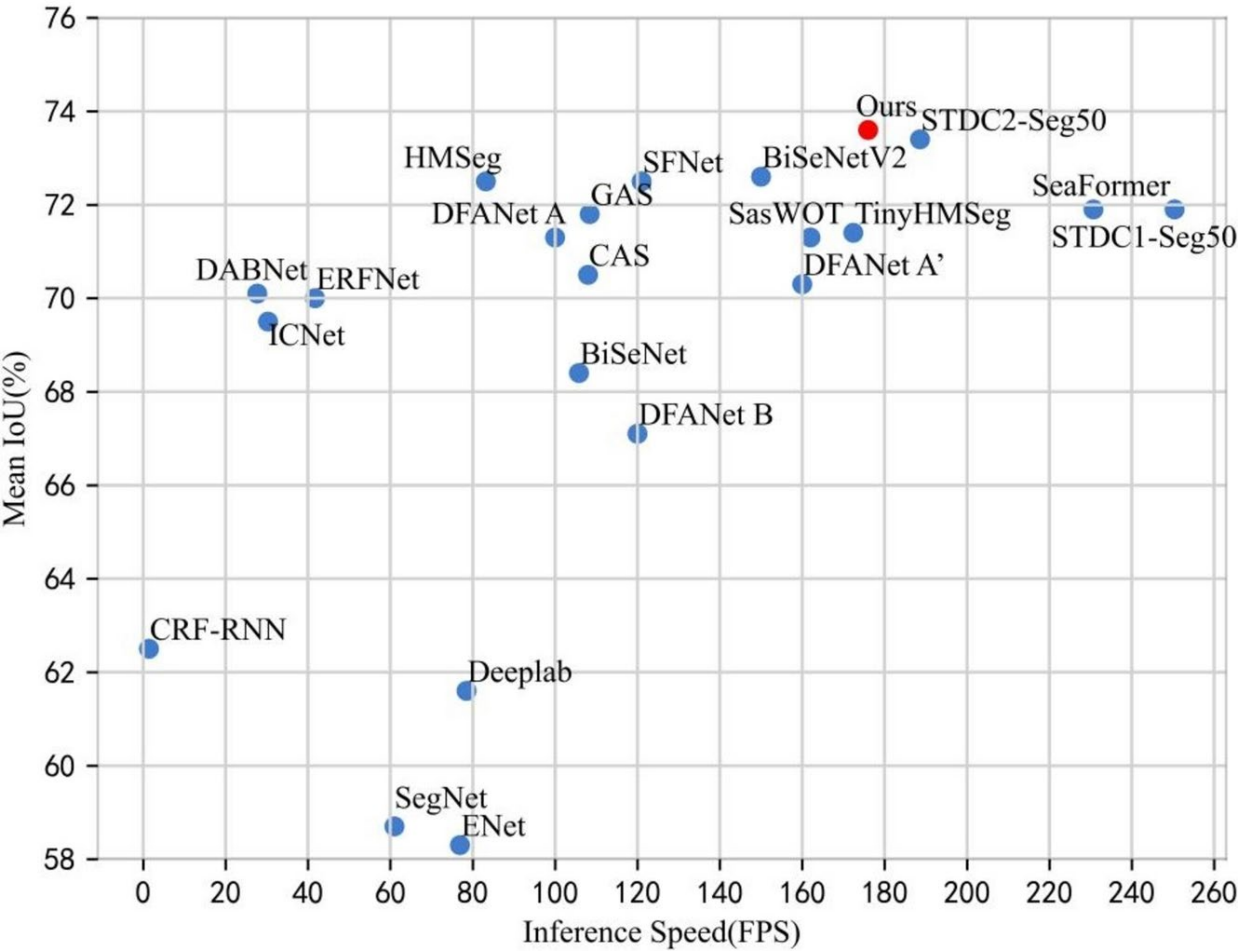
Speed-Accuracy performance comparison on the Cityscapes test dataset. The red represents our method, while the other methods are blue. Our method achieves the excellent speed-accuracy trade-off.

We evaluated the performance of the proposed real-time semantic segmentation network on two datasets, Cityscapes and Camvid. In this chapter, we first introduce the data and some specific implementation details. Then, we conduct partial research and experiments on our proposed approach. All experiments mentioned above are performed on the Cityscapes dataset. Finally, we present the accuracy and efficiency results under different conditions and compare them with existing methods through experimental analysis. The speed-accuracy trade-off is illustrated in (Fig. 5).

### Dataset

#### Cityscapes

Cityscapes^35^ is one of the classic datasets for urban driving scenes, consisting of 5000 finely annotated images divided into training, validation, and test sets, with 2975, 500, and 1525 images, respectively. We utilize 19 classes for experimentation. The dataset has a resolution of 1024 × 2048, which is relatively large within the dataset. We only employ images with fine annotations to ensure a fair comparison with other methods.

#### Camvid

The cambridge-driving labeled video database (Camvid)^36^ is a high-resolution driving scene dataset. Its resolution is 960 × 720 and consists of 32 semantic categories, with 11 classes used for semantic segmentation experiments. The dataset comprises 701 frames extracted from video sequences, of which 367 are selected for training, 233 for validation, and 101 for testing.

### Experimental results

We utilize mini-batch stochastic gradient descent (SGD)^13^ with a momentum of 0.9 and a weight decay of 5e-4. Additionally, we employ a “poly” learning rate adjustment strategy in which the initial rate is multiplied by ${(1 - \frac{\text{iter}}{\text{max}\_\text{iter}})}^{\text{power}}$.

We set the initial learning rate to 0.01 with a power value 0.9 for the Cityscapes and Camvid datasets, with training batch sizes of 8 and 6, respectively. We set max_iter to 80,000 times. Linear warmup^37^ training methods are applied to both datasets within the first 1000 iterations. All experiments are conducted using PyTorch-1.1 on NVIDIA GTX 1080 Ti GPU with CUDA 10.2 and batch size 1. Regarding data augmentation, we perform random horizontal flipping, cropping, and scaling on input images. For training on Cityscapes, cropping ranges from {0.125, 0.25, 0.375, 0.5, 0.625, 0.75, 0.875, 1.0, 1.125, 1.25, 1.375, 1.5}, with a crop resolution 1024 × 512. For training on Camvid, cropping ranges from {0.125, 0.25,0.375, 0.5, 0.625, 0.75, 0.875, 1.0, 1.125, 1.25, 1.375, 1.5, 1.625, 1.75, 1.875, 2.0, 2.125, 2.25, 2.375, 2.5}, with a crop resolution of 960 × 720.We use MIoU as an evaluation indicator, ${p}_{ij}$ means predicting class *i* to class *j*, which is false negative (FN), ${\text{p}}_{\text{ji}}$ means predicting class* j* to class* i*, which is false positive (FP), ${p}_{ii}$ means predicting class *i* to class *i*, which is true (TP),and the MIoU formula is as follows:

5 $$MIoU=\frac{1}{k+1}\sum_{i=0}^{k}\frac{{p}_{ii}}{{\sum }_{j=0}^{k}{p}_{ij}+{\sum }_{j=0}^{k}{p}_{ji}-{p}_{ii}}$$

### Ablation experiment

This section describes ablation experiments conducted to validate the effectiveness of our proposed method. We validate our approach based on the Cityscapes dataset in the ablation experiments. In Table 1, we compare the categories of our proposed method with those of other models.

**Table 1:** Classification results of urban landscape data sets by different methods.

Method	Roa	Sid	Bui	Wal	Fen	Pol	TLi	TSi	Sky	Veg
ENet	96.3	74.2	85.0	32.1	33.2	43.4	34.1	44.0	90.6	88.6
ERFNet	97.7	81.0	89.8	42.5	48.0	56.2	59.8	65.3	94.2	91.4
CGNet	95.9	73.9	89.9	43.9	46.0	52.9	55.9	63.8	94.1	91.7
EDANet	97.8	80.6	89.5	42.0	46.0	52.3	59.8	65.0	93.6	91.4
ESPNet	95.7	73.3	86.6	32.8	36.4	47.0	46.9	55.4	92.5	89.8
FSCNN	97.4	77.8	87.4	39.7	41.8	35.0	39.4	50.5	92.7	88.5
DABNet	97.8	80.7	90.2	47.9	48.1	56.4	61.8	67.0	94.3	92.0
FPENet	96.4	71.7	84.6	27.1	28.8	43.2	39.2	34.4	92.3	89.3
Our	99.5	85.6	93.1	55.8	54.1	60.8	67.2	70.1	97.6	96.1

Method	Bic		Mot		Ter		Ped		Tru		Car		Bus		Tra		Rid		mIoU	
ENet	55.4		38.8		61.4		65.5		36.9		90.6		50.5		48.1		38.4		58.3	
ERFNet	61.7		47.3		68.2		76.8		50.8		92.8		60.1		51.8		57.1		68.0	
CGNet	60.9		41.1		68.3		76.7		41.3		91.3		55.9		32.8		54.2		64.8	
EDANet	64.0		50.4		68.7		75.7		40.9		92.4		58.7		56.0		54.3		67.3	
ESPNet	54.9		36.4		66.0		68.5		40.0		89.9		47.7		40.7		45.9		60.3	
FSCNN	52.6		40.9		63.3		65.7		57.0		91.0		57.0		70.3		46.4		62.8	
DABNet	66.8		50.4		69.5		80.3		46.0		93.7		57.1		35.0		59.2		68.1	
FPENet	54.5		29.1		61.3		68.1		29.1		89.8		38.9		27.5		42.7		55.2	
Our	71.5		56.5		76.2		84.7		51.6		97.7		71.9		44.6		63.8		73.6	

### The initial value of the canny operator

Both Cityscape and Camvid datasets contain a large number of urban landscape images, which contain rich structural information and details. The edge detection algorithm can extract fine-grained features in images, such as borders, contours and textures. These features are very important for semantic segmentation tasks, especially when dealing with complex urban environments. Fine-grained features help the model to locate and segment objects of different categories more accurately. As multiple objects are often closely linked in Cityscape and Camvid data sets, the accuracy of boundary information is crucial for the correct segmentation of these objects. The edge detection algorithm can provide clear boundary information to help distinguish neighboring objects better. Edge detection algorithms usually do not require much computing resources, and can provide useful feature information without significantly increasing computing resources.

**Fig. 6:**
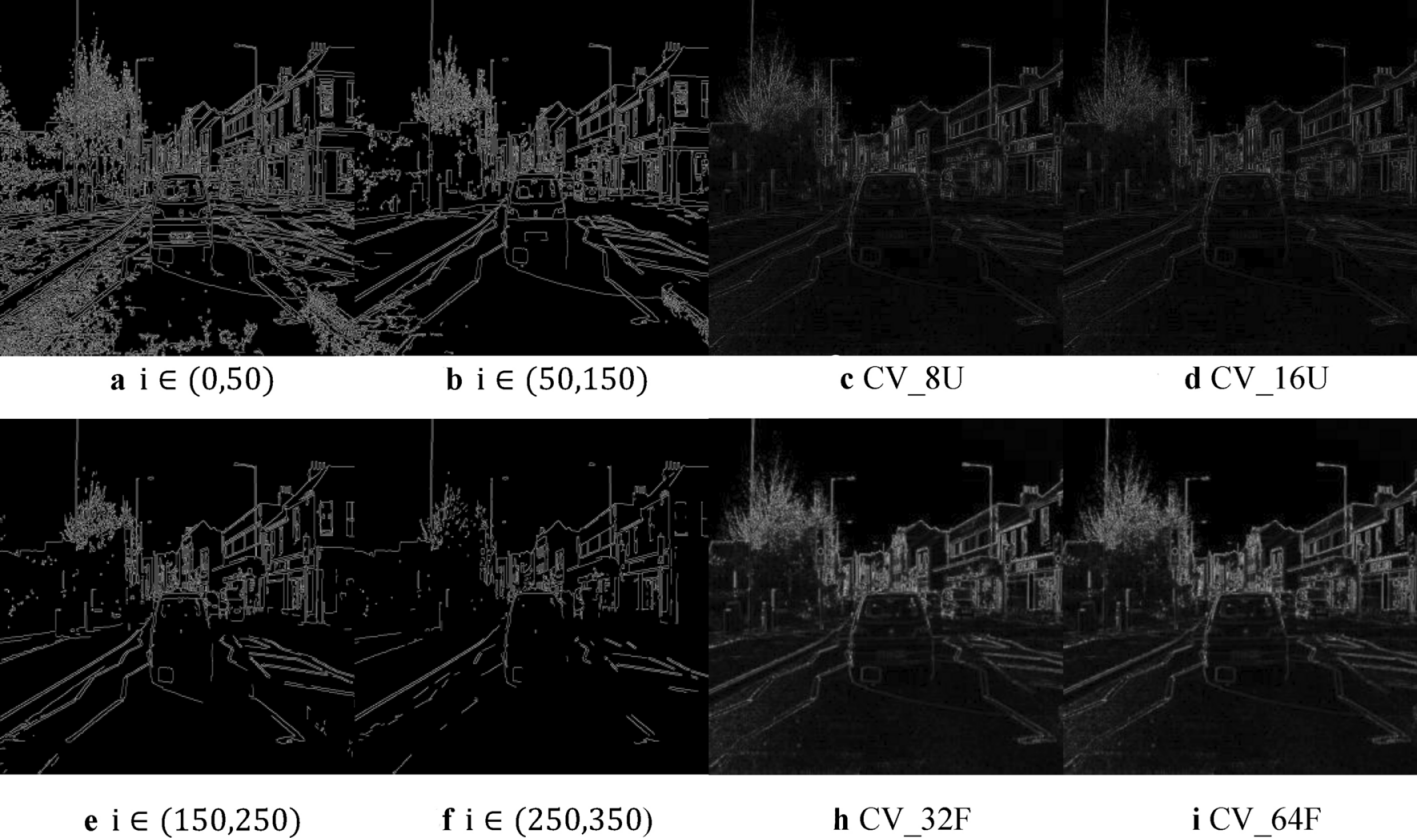
Visualization of canny operators and laplacian operators. (**a**,**b**,**e**,**f**) compare operators i in canny. (**c**,**d**,**h**), and (**i**) are comparisons of different ddepthi in laplacian.

In traditional edge detection algorithms, the Laplacian operator used by STDC Net is suitable when it only cares about the position of the edge and does not consider the grey difference of the surrounding pixels. Still, it is sensitive to noise and only applicable to noiseless images. The Canny operator we choose is not easily disturbed by noise and can detect the true weak edge. A visualization comparison between the Canny operator and the Laplacian operator shows that the Canny operator can detect more edge details. The core idea of Canny is to preserve the most obvious edge parts of the local area and suppress those edges that are not the most obvious, so as to make the final edge picture more clear and accurate. If the threshold of I is too high, some important edge information may be missed, as shown in Fig. 6 for e and f. If it is too low, too much noise may be detected, as shown in (Fig. 6a). The setting of threshold also affects the overall performance of the model, which is mainly reflected in the accuracy.

For specific implementation, we first set up the Canny module. After that, the feature graphs of 1/8 and 1/16 are Canny processed in the ContextPath part, and then ConvBNReLU operation is performed to maintain the standardization of the features. The “sum” operation is performed on the edge-processed 1/8 feature graph and the 1/16 feature graph. Finally, the processed image is up-sampled and the ContextPath 1/16 feature graph is “sum” operation.

As shown in (Table 1). Due to the requirement of real-time, we did not choose to add Canny anywhere, we chose to add it at 1/8 and 1/16. Based on the experimental results in Table 2, we ultimately obtain a range of {50,150}.

**Table 2:** Comparison of different canny operators on the Cityscapes val dataset. The size of the edge input is between 1/8 and 1/16 of the original input.

Canny operator i	Resolution	mIoU(%)	FPS
0–50	512 × 1024	71.0	176.3
50–150	512 × 1024	73.6	176.3
150–250	512 × 1024	71.4	176.3
250–350	512 × 1024	70.3	176.3

### Effectiveness of hybrid feature refinement module

**Fig. 7:**
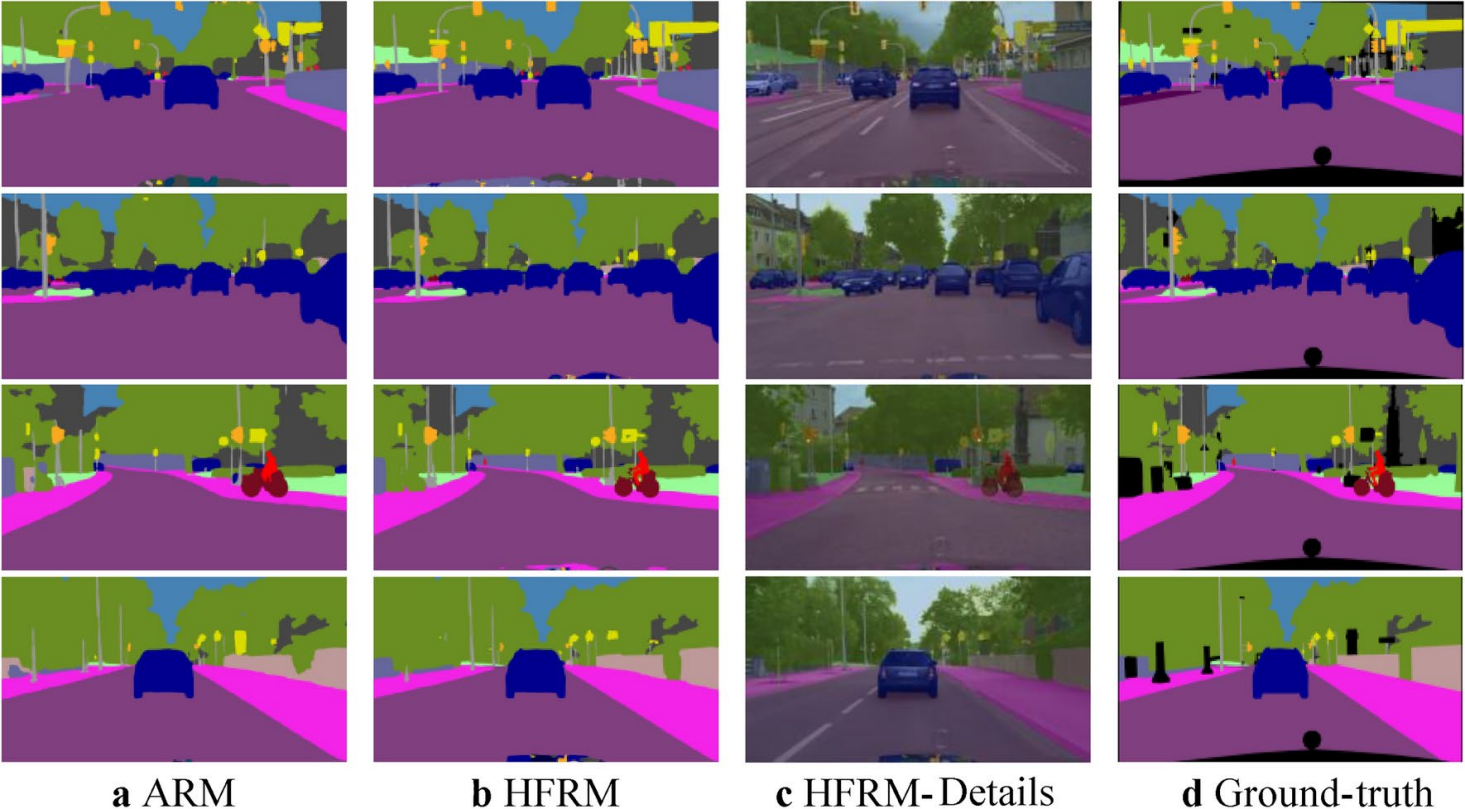
Visual comparison of our model on the Cityscapes dataset. HFRM denotes our proposed hybrid feature refinement module. The first column (**a**) is the ARM feature map. (**b**) and (**c**) are HFRM ground feature maps and details. (**d**) is the ground truth of input images.

We devised a hybrid feature refinement module (HFRM) to refine feature maps better. This module encodes output features into vectors by combining improved channel and spatial attention. To verify the effectiveness of our proposed method, we performed ablation experiments on the ARM^7^ module and the HFRM module. As shown in Fig. 7, edge parts and small objects can be detected more accurately using HFRM features than ARM features.

**Fig. 8:**
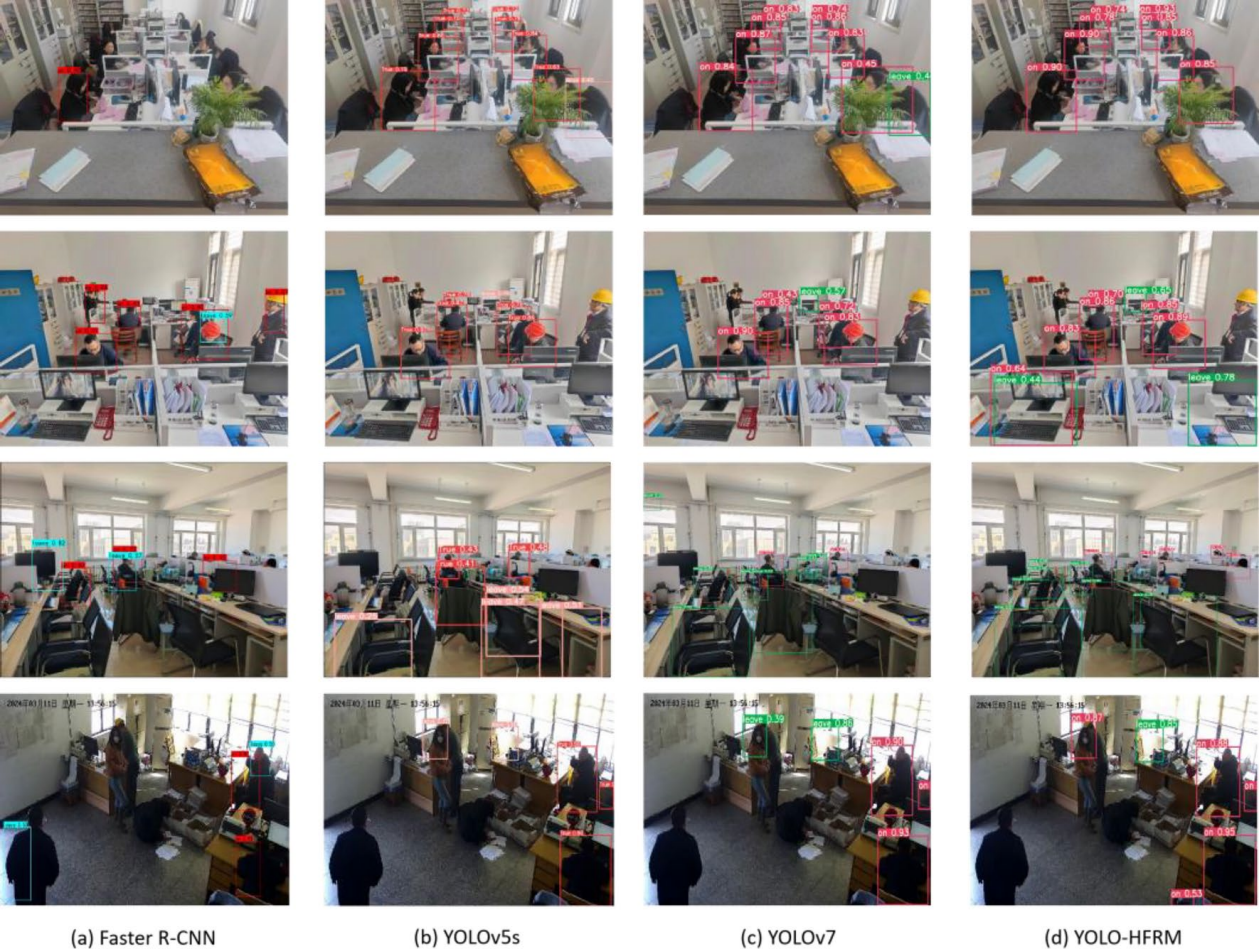
Detection results of YOLO-HFRM compared to other network models.

To verify the effect of HFRM, we selected a new data set for experiment. The data set has the characteristics of complex background and small personnel target. The new data set comes from our use of cameras for live photography and factory monitoring. We apply the HFRM module to YoloV7, and the category we detect is: personnel leaving the post detection. We set the image size to 640 × 640, the Epoch to 50, and the Batch size to 16. As shown in Fig. 8, the YOLO-HFRM demonstrated greater accuracy in complex backgrounds and small-target scenarios, especially in the case of dense blocks to identify people leaving. In contrast, R-CNN and YOLOv5s experienced many missed detections and false positives due to their limited feature extraction capabilities. YOLO-HFRM successfully detected targets with higher confidence scores compared to the baseline model YOLOv7. In terms of structure, since we designed HFRM as a plug-and-play approach, we can use it with simple adjustments when we use it, which is convenient for us to apply it to other models and other data sets.

### Effectiveness of hybrid feature fusion module

We designed the HFFM module to fuse two different levels of feature maps. These feature maps consist of high-level features with semantic information and low-level features with spatial information.

HFFM makes use of both channel attention and spatial attention dimensions. HFFM captures important information in feature maps more comprehensively through average pooling and maximum pooling operations. Moreover, the design of HFFM is simple and efficient, and does not require a lot of extra parameters and computation. This lightweight design allows HFFM to be integrated into most existing network frameworks. The Cityscapes dataset contains complex urban scenes in which objects vary widely in size, shape, and texture. HFFM is able to effectively extract and enhance key features in this variable environment. This makes HFFM perform well in the Cityscape semantic segmentation task, outperforming other single-attention mechanisms.

As shown in Table 3, the gap in test results highlights the significance of the HFFM module.

**Table 3:** Detailed performance comparison of each module in our proposed. We compared our module to the modules in Bisenet.

Method	mIoU(%)
CP + SP(FFM)	67.4
CP + SP(FFM) + ARM	68.7
Ours (HFFM)	69.4
Ours (HFRM + HFFM)	72.7
HFRM + HFFM + C	73.6

CP, SP, *ARM* attention refinement module, *FFM* feature fusion modules belong to BiSeNet. *HFRM* Hybrid feature refinement module, *HFFM* hybrid feature fusion module, *C* edge input.

Since the C supports the HFRM, we did not do a separate experimenter.

### Results on Cityscapes

**Fig. 9:**
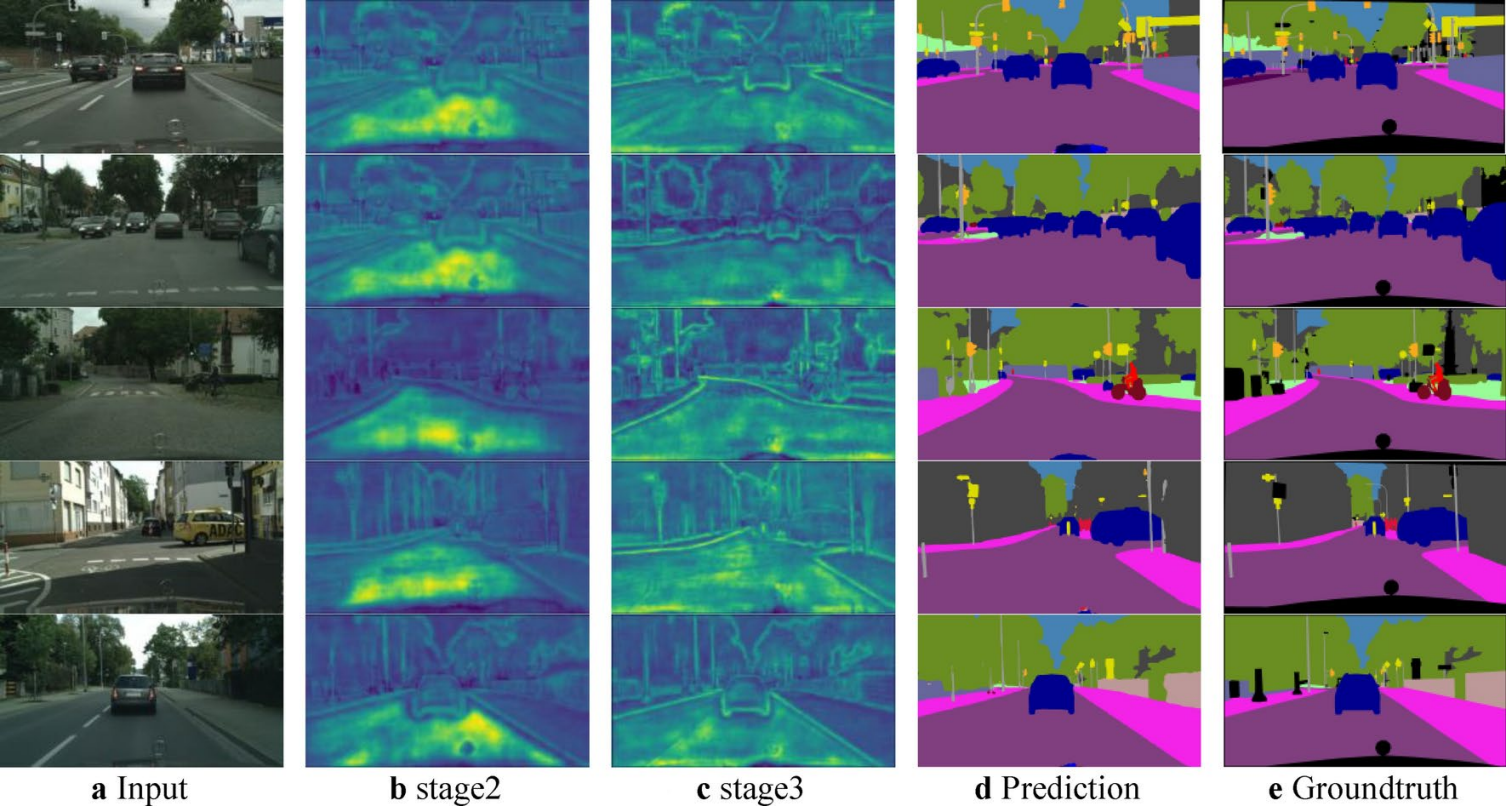
Visual comparison of our detail guidance on Cityscapes val set. The first row (**a**) shows the input images. (**b**,**c**) illustrate the heatmap without and with attention mechanisms. (**d**) demonstrate the predictions with the attention mechanism. (**e**) is the ground truth of input images.

We first visualize the heatmap of the feature map of Stage 3, as shown in (Fig. 9). The third-stage features with attentional mechanisms encode more spatial information than the second-stage features without attentional mechanisms. As a result, the final predictions for small objects and boundaries are more precise. As shown in Table 4, we present our proposed method’s segmentation accuracy and inference speed on the Cityscapes dataset. Our approach achieves the optimal speed-accuracy trade-off in terms of both accuracy and real-time performance. We accomplish a mIoU of 73.6% at 176 FPS. Compared to SFNet^38^ and STDC1-Seg50, we improve accuracy by 1.1 and 1.7 percentage points. Compared to the performance-leading BiSeNetV2^39^, we achieved a 1.0% improvement in accuracy.

**Table 4:** Accuracy and speed comparison on Cityscapes. No indicates the method does not have a backbone.

Model	Resolution	Backbone	mIoU(%)	FPS
Non real-time				
SegNet^16^	360 $\times$ 640	VGG16	58.7	61.0
CRF-RNN^40^	512 $\times$ 1024	VGG16	62.5	1.4
CAS^41^	768 $\times$ 1536	No	70.5	108.0
SFNet^38^	1024 $\times$ 2048	DF1	72.5	121.0
Real-time				
Enet^3^	512 $\times$ 1024	No	58.3	76.9
DeepLab^42^	512 $\times$ 1024	VGG16	61.6	78.5
DFANet B^43^	1024 $\times$ 1024	Xception B	67.1	120.0
BiSeNet^7^	768 $\times$ 1536	Xception39	68.4	105.8
ICNet^44^	1024 $\times$ 2048	PSPNet50	69.5	30.3
ERFNet^20^	512 $\times$ 1024	No	70.0	41.7
DABNet^45^	1024 $\times$ 2048	No	70.1	27.7
DFANet A’^43^	512 $\times$ 1024	Xception A	70.3	160.0
SasWOT^46^	1024 $\times$ 2048	resnet101	71.3	162.0
TinyHMSeg^47^	768 $\times$ 1536	No	71.4	172.4
GAS^48^	769 $\times$ 1537	No	71.8	108.4
SeaFormer^24^	512 $\times$ 1024	SeaFormer-B	71.9	230.7
STDC1-Seg50^10^	512 $\times$ 1024	STDC1	71.9	250.4
HMSeg^47^	768 $\times$ 1536	No	72.5	83.2
BiSeNetV2^39^	512 $\times$ 1024	No	72.6	150.0
SCTNet-S-Seg50^49^	512 $\times$ 1024	No	72.6	450.5
STDC2-Seg50^10^	768 $\times$ 1536	STDC2	73.4	188.6
Ours	512 $\times$ 1024	STDC2	73.6	176.0

### Results on Camvid

We evaluated our method on the Camvid dataset. We use Table 5 to compare the other methods in terms of complexity. Since both HFRM and HFFM proposed by us are linear levels, the complexity is $o\left(n\right)$. The attention proposed by DANet and Transformer is ${\text{o}\left(\text{n}\right)}^{2}$, which further verifies the real-time performance of our proposed method. Table 6 presents an experimental result compared to other methods. With an input size of 720 × 960, our proposed Model achieves a mIoU of 70.0% and 193.2FPS. Compared to other methods, our approach significantly improves performance and speed. This validates the effectiveness of our proposed method.

**Table 5:** Complexity comparison.

Model architecture	Complexity
Non-local net	${o\left(n\right)}^{2}$
Transformer	${o\left(n\right)}^{2}$
Sparse tansformer	$o\left(n\sqrt{n}\right)$
DANet	${o\left(n\right)}^{2}$
Our	$o\left(n\right)$

**Table 6:** Accuracy and speed comparison with other state-of-the-art methods on Camvid. No indicates the method does not have a backbone.

Model	Resolution	Backbone	mIoU(%)	FPS
Non real-time				
ESPNet^50^	720 $\times$ 960	No	55.6	112.0
SegNet^16^	720 $\times$ 960	VGG16	60.1	4.6
RTA^51^	720 $\times$ 960	VGG16	62.5	0.2
PSPNet^52^	720 $\times$ 960	ResNet50	69.1	5.4
Real-time				
Enet^3^	720 $\times$ 960	No	51.3	61.2
DFANet B^43^	720 $\times$ 960	Xception B	59.3	160.0
Deeplab^42^	720 $\times$ 960	VGG16	61.6	4.9
DFANet A^43^	720 $\times$ 960	Xception A	64.7	120.0
BiSeNet^7^	720 $\times$ 960	Xception39	65.6	175.0
STDC1-Seg^10^	720 $\times$ 960	STDC 1	67.5	50.3
Ours	720 $\times$ 960	STDC 2	70.0	146.3

## Conclusion

In this paper, we reexamine the current incorporation of attention mechanisms into feature processing. While building upon the foundation, we highly acknowledge the dual architecture of BiSeNet and the encoder of STDC Net. However, the additional spatial path in BiSeNet affects inference efficiency; thus, adopting STDC as the backbone significantly alleviates this issue. We have redesigned the modules within the network. The original ARM and FFM in BiSeNet lack low-level spatial information. Leveraging lightweight concepts of channel and spatial attention, we introduce HFRM and HFFM. HFRM refines feature mappings more finely, aided by edge detection methods. HFFM effectively integrates channel and spatial attention to fuse feature maps at different levels. These methods collectively enhance the accuracy of semantic segmentation results. Considering inference speed, the proposed architecture balances speed and accuracy well. Extensive experiments and visualization results demonstrate the feasibility of our proposed approach.

## Data Availability

The Cityscapes data for this study can be found in the [https://www.cityscapes-dataset.com/]. The Camvid data for this study can be found in the [https://www.kaggle.com/datasets/carlolepelaars/camvid]. Other data in this article,the data are available from the corresponding author on reasonable request.
